# Factors Influencing Health Facility Delivery in Predominantly Rural Communities across the Three Ecological Zones in Ghana: A Cross-Sectional Study

**DOI:** 10.1371/journal.pone.0152235

**Published:** 2016-03-31

**Authors:** Yeetey Akpe Kwesi Enuameh, Sumiyo Okawa, Kwaku Poku Asante, Kimiyo Kikuchi, Emmanuel Mahama, Evelyn Ansah, Charlotte Tawiah, Kwame Adjei, Akira Shibanuma, Keiko Nanishi, Francis Yeji, Enoch Oti Agyekum, Junko Yasuoka, Margaret Gyapong, Abraham Rexford Oduro, Gloria Quansah Asare, Abraham Hodgson, Masamine Jimba, Seth Owusu-Agyei, Ghana EMBRACE Implementation Research Project Team

**Affiliations:** 1 Kintampo Health Research Centre, P.O. Box 200, Kintampo, Brong-Ahafo, Ghana; 2 Department of Community and Global Health, Graduate School of Medicine, The University of Tokyo, 7-3-1 Hongo, Bunkyo-ku, Tokyo, 113–0033, Tokyo, Japan; 3 Research and Development Division, Ghana Health Service, P. O. Box MB 190, Accra, Ghana; 4 Navrongo Health Research Centre, P.O. Box 114, Navrongo, Upper-East, Ghana; 5 System Science Consultants, Tokyo, Japan; 6 Dodowa Health Research Centre, P.O. Box DD1, Dodowa, Greater Accra, Ghana; 7 Ghana Health Service Headquarters, Private Mail Bag, Ministries, Accra, Ghana; National Institute of Health, ITALY

## Abstract

**Background:**

Maternal and neonatal mortality indicators remain high in Ghana and other sub-Saharan African countries. Both maternal and neonatal health outcomes improve when skilled personnel provide delivery services within health facilities. Determinants of delivery location are crucial to promoting health facility deliveries, but little research has been done on this issue in Ghana. This study explored factors influencing delivery location in predominantly rural communities in Ghana.

**Methods:**

Data were collected from 1,500 women aged 15–49 years with live or stillbirths that occurred between January 2011 and April 2013. This was done within the three sites operating Health and Demographic Surveillance Systems, i.e., the Dodowa (Greater Accra Region), Kintampo (Brong Ahafo Region), and Navrongo (Upper-East Region) Health Research Centers in Ghana. Multivariable logistic regression was used to identify the determinants of delivery location, controlling for covariates that were statistically significant in univariable regression models.

**Results:**

Of 1,497 women included in the analysis, 75.6% of them selected health facilities as their delivery location. After adjusting for confounders, the following factors were associated with health facility delivery across all three sites: healthcare provider’s influence on deciding health facility delivery, (AOR = 13.47; 95% CI 5.96–30.48), place of residence (AOR = 4.49; 95% CI 1.14–17.68), possession of a valid health insurance card (AOR = 1.90; 95% CI 1.29–2.81), and socio-economic status measured by wealth quintiles (AOR = 2.83; 95% CI 1.43–5.60).

**Conclusion:**

In addition to known factors such as place of residence, socio-economic status, and possession of valid health insurance, this study identified one more factor associated with health facility delivery: healthcare provider’s influence. Ensuring care provider’s counseling of clients could improve the uptake of health facility delivery in rural communities in Ghana.

## Background

Maternal and neonatal mortality indicators remain high for most countries in sub-Saharan Africa, including Ghana. The global maternal mortality rate was 210 per 100,000 live births in 2013 [1]. Of the 289,000 maternal deaths globally recorded in 2013, sub-Saharan Africa accounted for 179,000 (62%) [1]. The maternal mortality rate in Ghana currently stands at 380 deaths per 100,000 live births, as compared to the 2015 Millennium Development Goal target of 185 per 100,000 [2,3].

As of 2013, neonatal mortality rate was 20 per 1,000 live births worldwide, and 31 per 1,000 live births in sub-Saharan Africa [2]. Of the 2,763,000 neonatal deaths recorded globally in 2013, sub-Saharan Africa contributed 1,066,000 (39%). As of 2011, the neonatal mortality rate in Ghana was 32 deaths per 1,000 live births [3]. Ghana’s Millennium Development Goal 4 target for under-5 mortality is 39.9 deaths per 1,000 live births, but at the 2010 Population and Housing Census, it was 59 deaths per 1,000 [4]. Ghana was not able to achieve its Millennium Development Goals 4 and 5 of reducing child mortality and improving maternal health respectively, by 2015 [3].

As the delivery process can result in unexpected complications [5], health facility delivery is crucial. About three quarters of all maternal [6] and most perinatal [7] deaths occur during delivery and in the immediate post-partum period. Preventable causes, such as post-partum hemorrhage, sepsis, obstructed labor, and eclampsia, are known to contribute to maternal mortality significantly [7]. When a woman delivers with assistance from a skilled birth attendant (SBA) in a health facility, she can receive basic obstetric care, neonatal care, and emergency care—resulting in improved, maternal and neonatal health outcomes [3]. Health facility delivery could facilitate postpartum care of the mother and neonate. Such care includes family planning, vaccination, and nutrition services [8]. However, health facility delivery rates remain low in most low- and middle- income countries [9–12].

Several factors influence the location where women deliver globally. Such factors include place of residence, family decision-making regarding place of delivery, ANC attendance, socio-economic status (SES), trimester of pregnancy, age of woman, parity, transport, placenta disposal, delivery position, complication at last delivery, age of pregnancy, levels of education of the woman and her partner, and valid health insurance [5, 9–22].

In Ghana, health facility deliveries have increased to 73% from 42% over the last two decades [5]. However, close to 30% of deliveries occur in homes [6, 23]. Two studies explored determinants of health facility deliveries in Ghana [13, 19]. The first study identified SES and women's education as linked to health facility delivery [13]. The other study showed that maternal and partner education, and SES were associated with SBA delivery [19]. These studies, however, did not comprehensively explore other factors that could influence health facility delivery. This study identified some other factors that influence the place of delivery for pregnant women across diverse ecological zones in Ghana.

## Methods

### Study design

This cross-sectional study was conducted in three predominantly rural areas of Ghana from July to September 2013. The study is a part of the Ghana Ensure Mothers and Babies Regular Access to Care (EMBRACE) Implementation Research program, which aimed at strengthening the continuum of care for maternal, newborn and child health (MNCH) and subsequently improving MNCH outcomes [24].

### Study setting

**Fig 1:**
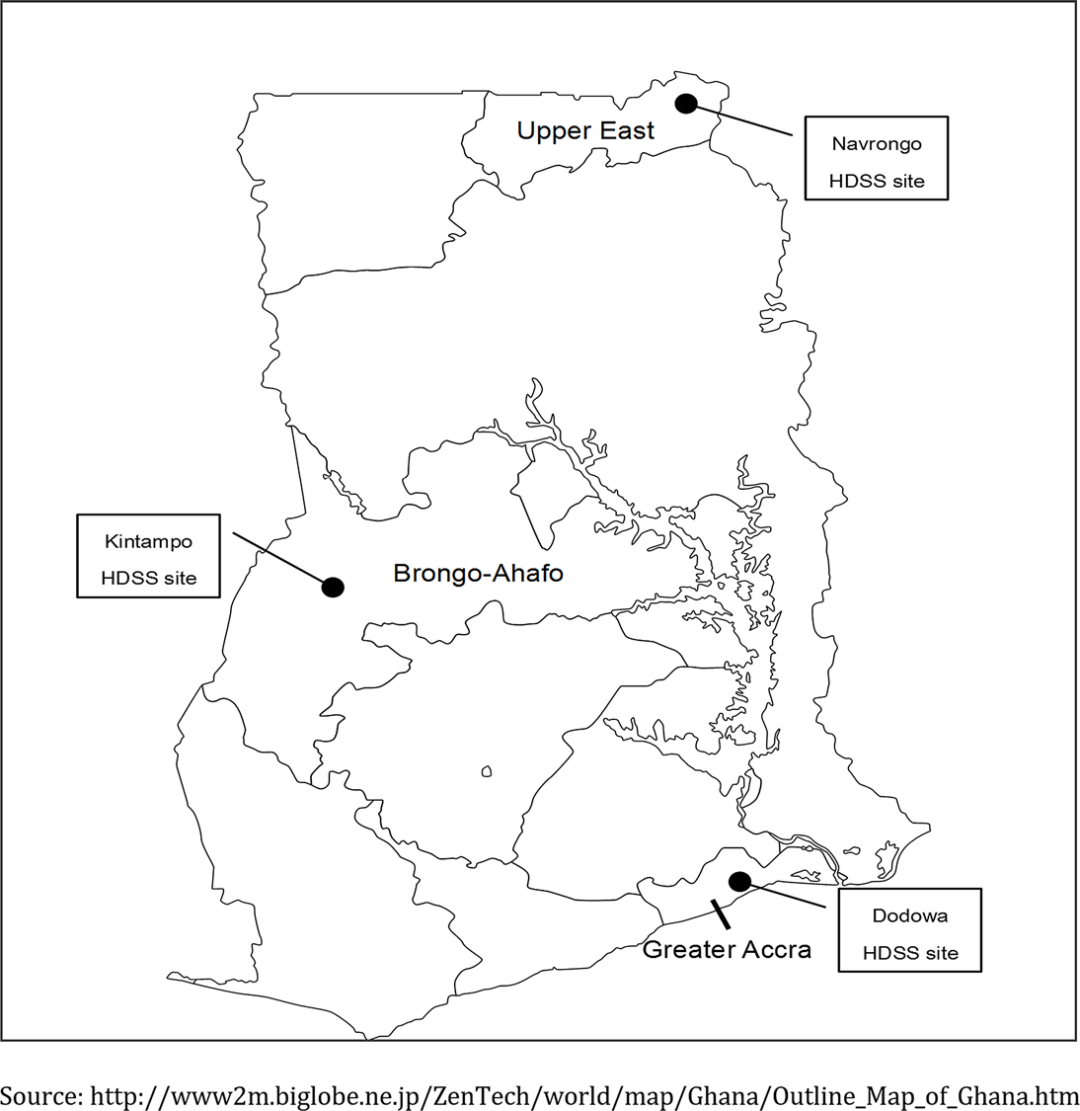
Map of Ghana showing the geographical location of the study sites.

Dodowa, Kintampo, and Navrongo were selected as study sites. The sites have diverse ecological and health delivery systems (Fig 1, Table 1). Each study site has a Health and Demographic Surveillance System (HDSS), which collects longitudinal data on population risks, exposures, and outcomes [25]. Dodowa is located in the southern part of Ghana. Its HDSS covers the Shai-Osudoku and the Ningo Prampram districts [26]. Kintampo is located in the central part of the country. The Kintampo HDSS covers the Kintampo North Municipality and the Kintampo South District [27]. Finally Navrongo, located in the northern part of the country, has its HDSS covering the Kassena-Nankana East and West Districts [28]. The Community-based Health Planning and Services (CHPS) program was introduced to deprived communities in Ghana in 2002 [29, 30], to facilitate geographical equity in MNCH care delivery. CHPS is most developed in Navrongo [31], followed by Dodowa and Kintampo. Community Health Officers (CHOs) in the CHPS compounds have midwifery skills in Navrongo and Dodowa, whereas those in Kintampo do not have these skills.

**Table 1:** Population indices of the study sites.

Indicators	Dodowa^1^ (Southern)	Kintampo^2^ (Central)	Navrongo^3^ (Northern)
Total resident population	111,976	148,124	157,629
Fertility rate	2.7	4.4	3.5
Crude birth rate (births/1000 population)	23.5	33.1	25.1
Neonatal mortality (deaths/1000 live births)	8.8	27.9	12.4
Infant mortality (deaths/1000 live births)	19.8	46.5	27.5

^1^ Dodowa Health and Demographic Surveillance System, 2011 [26]

^2^ Kintampo Health and Demographic Surveillance System, January 2013 [32]

^3^ Navrongo Health and Demographic Surveillance System, January 2013 [33]

### Study population

The women were recruited according to the following criteria: be aged 15 to 49 years old, should have had a live or stillbirth between January 2011 and April 2013, and be resident in the study area at the time of the study. If women had more than one pregnancy and delivery over the study period, the most recent pregnancy information was collected. Exclusion criteria were those who had an abortion or a miscarriage during the period of the study.

### Sampling

Women involved in the study were sampled from the HDSS databases of the three sites. Two-stage random sampling was used to select 22 primary sampling units, from which 1,500 women were recruited (500 from each site). The zone or sub-district was used as the primary sampling unit depending on the study site. The zone as a unit of population representation was developed by the Navrongo HDSS. A sub-district is the lowest unit in the local government structure of Ghana after the Regions and Districts [34].

### Data collection

The questionnaire for the study was developed based on the 2007 Ghana Maternal Health Survey [35] and the National Safe Motherhood Service protocol [36]. The questionnaire covered background characteristics, antenatal history, socio-economic status (SES), services women received during pregnancy, and delivery. The questionnaire was reviewed by Ghanaian experts in the field of MNCH. The questionnaire was finalized based on the findings from pretesting. Additionally, data on ethnicity, religion, and household assets were obtained from the HDSS datasets of the three sites. During data collection, trained field workers administered the questionnaires through face-to-face interviews with women.

### Data management, measurement and analysis

Data were double entered into Microsoft Foxpro version 9. Verification and consistency checks were performed to ensure completeness of the data. Data were transferred to the Statistical Package for Social Sciences (SPSS) version 22 [37] for statistical analysis.

The dependent variable was venue of the last delivery (i.e. health facility delivery or non-health facility delivery). Health facilities included public hospitals/polyclinics, private hospitals/clinics/maternity homes, health centers and CHO offices/CHPS compounds/community clinics. Locations outside of health facilities (non-health facility) included traditional birth attendants’ homes, on the way to the health facility, and the women’s homes.

Independent variables were categorized as background characteristics, antenatal history, and socio-economic characteristics. Background characteristics include mothers’ age, partners’ age, current marital status, ethnicity, religion, mothers’ educational attainment, and partners’ educational attainment. Antenatal history consists of number of births at last delivery, frequency of ANC attendance, desire for pregnancy, and education on danger signs of pregnancy during ANC. Socio-economic characteristics include site of residence, person who influenced the decision on place of delivery, possession of valid health insurance card, money readily available to seek healthcare, and SES. Assets used in the generation of wealth quintiles for SES included 19 items. They were ownership of land, house, wall type, roof type, water source, cooking fuel, available electrical power, television, radio, bicycle, bed-net, toilet facility, type of roofing on the building, motor bike, car, cell/landline phone, sewing machine, gas/electric cooker, and fridge/freezer. The wealth quintiles were created based on the methods used by the Demographic and Health Surveys [38].

Descriptive analysis was performed to summarize the background characteristics of the women. Logistic regression was run to identify determinants of health facility delivery at all and individual sites respectively. For all sites, univariable logistic regression was performed to determine the associations between health facility delivery and each independent variable. Multivariable logistic regression was further used to adjust for covariates that were statistically significant in the univariable regression models. For individual sites, univariable and multivariable logistic regression followed a similar method as that used for all the sites. A two-sided p-value of less than 0.05 was considered as statistically significant.

### Ethical considerations

Ahead of implementation of the study, ethical approval was obtained from the Dodowa Health Research Centre Institutional Review Board, the Kintampo Health Research Centre Institutional Ethics Committee, the Navrongo Health Research Centre Institutional Review Board, the Ghana Health Service Ethics Review Committee, and the Research Ethics Committee of The University of Tokyo, Japan.

Prior to participating in the study, all women endorsed a written informed consent form. Persons below 18 years of age had the consent form signed by their parents or caregivers ahead of taking part in the study. Copies of the consent forms were stored in secured data banks of the three health research centers. Confidentiality of the women was strictly enforced.

## Results

### Background characteristics of study participants

Data from 1,497 respondents were analyzed (Dodowa: 500, Kintampo: 500, Navrongo: 497). Table 2 provides the background characteristics of the women taking part in the study. Up to 1,131 (75.6%) women delivered in health facilities. Half were between the ages of 20 and 29 (49.9%), and 33.8% were in the 30–39-year age group. Partners of the women were predominantly aged 30–39 years (37.7%). The majority of the women were married (60.8%), and about a quarter (26.4%) were cohabiting. Forty-three percent of the women were from the northern tribes. The dominant religions were Christianity (52.8%) and Islam (14.0%). Thirty-nine percent of women and 28.9% of their partners had no educational experience.

**Table 2:** Background characteristics of study participants (N = 1,497).

Characteristics	Non-health facility delivery (n = 366)		Health facility Delivery (n = 1,131)		Overall (n = 1,497)	
	n	%	n	%	n	%
**Age of mother**						
10–19	30	(8.2)	100	(8.8)	130	(8.7)
20–29	177	(48.4)	570	(50.4)	747	(49.9)
30–39	129	(35.2)	377	(33.3)	506	(33.8)
40–49	21	(5.7)	76	(6.7)	97	(6.5)
Don't know	9	(2.5)	8	(0.7)	17	(1.1)
**Age of partner**						
≤ 29	60	(16.4)	242	(21.4)	302	(20.2)
30–39	87	(23.8)	387	(34.2)	471	(37.7)
40–49	43	(11.7)	167	(14.8)	210	(14.0)
≥ 50	12	(3.3)	51	(4.5)	63	(4.2)
No partner/don’t know	164	(44.8)	284	(25.1)	448	(29.9)
**Marital status**						
Married	191	(52.2)	719	(63.6)	910	(60.8)
Cohabiting	123	(33.6)	272	(24.0)	395	(26.4)
Divorced/separated/widowed	11	(3.0)	37	(3.3)	48	(3.2)
Never married	41	(11.2)	103	(9.1)	144	(9.6)
**Ethnicity**						
Northern tribes	140	(38.3)	503	(44.5)	643	(43.0)
Akan	101	(27.6)	253	(22.4)	354	(23.6)
Ga/Adangbe/Ewe	41	(11.2)	195	(17.2)	236	(15.8)
Others	69	(18.9)	120	(10.6)	189	(12.6)
Missing	15	(4.1)	60	(5.3)	75	(5.0)
**Religion**						
Christian	192	(52.5)	598	(52.9)	790	(52.8)
Islam	77	(21.0)	132	(11.7)	209	(14.0)
Traditional	66	(18.0)	286	(25.3)	352	(23.0)
Other	17	(4.6)	58	(5.1)	75	(5.0)
Missing	14	(3.8)	57	(5.0)	71	(4.7)
**Educational attainment of mother**						
None	199	(54.4)	385	(34.0)	584	(39.0)
Primary	90	(24.6)	254	(22.5)	344	(23.0)
Middle/JSS^a^/JHS^b^	68	(18.6)	356	(31.5)	424	(28.3)
Secondary/SSS^c^/SHS^d^/Tech^e^/Voc^f^	8	(2.2)	103	(9.1)	111	(7.4)
Tertiary and above	1	(0.3)	33	(2.9)	34	(2.3)
**Educational attainment of partner**						
None	143	(39.1)	290	(25.6)	433	(28.9)
Primary	54	(14.8)	140	(12.4)	194	(13.0)
Middle/JSS^a^/JHS^b^	93	(25.4)	326	(28.8)	419	(28.0)
Secondary/SSS^c^/SHS^d^/Tech^e^/Voc^f^	32	(8.7)	189	(16.7)	221	(14.8)
Tertiary and above	8	(2.2)	95	(8.4)	103	(6.9)
Not applicable/don’t know	36	(9.8)	91	(8.0)	127	(8.5)

^a^JSS: Junior Secondary School

^b^JHS: Junior High School

^c^SSS: Senior Secondary School

^d^SHS: Senior High School

^e^Tech: Technical School

^f^Voc: Vocational School

### Antenatal history

Table 3 gives an overview of the antenatal history of the women in the study. Majority of women (68.6%) had four births or less at the time of data collection. The majority of mothers (86.1%) had four or more ANC attendances. Almost three quarters of women (72.4%) were educated on danger signs of pregnancies during ANC attendance.

**Table 3:** Antenatal history (N = 1,497).

Characteristics	Non-health facility delivery (n = 366)		Health facility delivery (n = 1,131)		Overall (n = 1,497)	
	n	%	n	%	n	%
**Number of births at last delivery**						
≤ 4 children	231	(63.1)	796	(70.4)	1,027	(68.6)
> 4 children	135	(36.9)	335	(29.6)	470	(31.4)
**ANC attendance**						
< 4 times	97	(26.5)	111	(9.8)	208	(13.9)
≥ 4 times	269	(73.5)	1,020	(90.2)	1,289	(86.1)
**Desire for pregnancy**						
Wanted at conception	197	(53.8)	678	(59.9)	875	(58.5)
Wanted later	129	(35.2)	352	(31.1)	481	(32.1)
Did not want at all	40	(10.9)	101	(8.9)	141	(9.4)
**Education on danger signs of pregnancy during ANC**						
Yes	239	(65.3)	845	(74.7)	1,084	(72.4)
No	113	(30.9)	275	(24.3)	388	(25.9)
Not applicable/don’t remember	14	(3.8)	11	(1.0)	25	(1.7)

### Socio-economic characteristics

Table 4 describes the socio-economic characteristics of the women. Women who delivered within health facilities went by automobile (41.1%), bicycle/tricycle/motorcycle (16.5%), or on foot (40.8%). Under half (46.3%) possessed valid health insurance card, and 43.5% had funds available at home to seek healthcare.

**Table 4:** Socio-economic characteristics (N = 1,497).

Characteristics	Non-health facility delivery (n = 366)		Health facility delivery (n = 1,131)		Overall (n = 1,497)	
	n	%	n	%	n	%
**Influence on women’s decision on delivery venue**						
Non-healthcare provider	358	(97.8)	763	(67.5)	1,121	(74.9)
Healthcare provider	8	(2.2)	368	(32.5)	376	(25.1)
**Means of transportation to delivery venue**						
On foot	343	(93.7)	268	(23.7)	611	(40.8)
Bicycle/tricycle/motorcycle	0	(0.0)	247	(21.8)	247	(16.5)
Taxi/public transport/private car	1	(0.3)	614	(54.3)	615	(41.1)
Not applicable*	22	(6.0	2	(0.2)	24	(1.6)
**Possess a valid health insurance card**						
No	112	(30.6)	284	(25.1)	396	(26.5)
Yes	107	(29.2)	588	(52.0)	695	(46.4)
Not applicable	147	(40.2)	259	(22.9)	406	(27.1)
**Money readily available in household to seek healthcare**						
No	215	(58.7)	610	(53.9)	825	(55.1)
Yes	144	(39.3)	507	(44.8)	651	(43.5)
Not applicable	7	(1.9)	14	(1.2)	21	(1.4)
**Wealth quintiles**						
Least wealthy	79	(21.6)	231	(20.4)	310	(20.7)
Less wealthy	92	(25.1)	198	(17.5)	290	(19.4)
Wealthy	109	(29.8)	189	(16.7)	298	(19.9)
Wealthier	66	(18.0)	234	(20.7)	300	(20.0)
Wealthiest	20	(5.5)	279	(24.7)	299	(20.0)

*Persons who did not walk, did not use any motorized means of transportation, or did not remember their means of transportation.

### Proportion of health facility delivery

At each study site, 75.8% of women from Dodowa, 61.6% from Kintampo, and 89.3% from Navrongo, respectively, delivered at health facilities (Table 5).

**Table 5:** Proportion of health facility delivery at all and individual sites (N = 1,497).

Site	Non-health facility delivery		Health facility delivery	
	n	%	n	%
All sites	366	(24.4)	1,131	(75.6)
Dodowa	121	(24.2)	379	(75.8)
Kintampo	192	(38.4)	308	(61.6)
Navrongo	53	(10.7)	444	(89.3)

### Determinants of health facility delivery across all sites

In the univariate analyses, 12 independent variables were associated with place of delivery. They were mother’s educational attainment, partner’s educational attainment, number of births at last delivery, marital status, ethnicity, religion, antenatal attendance, education on danger signs of pregnancy, site of residence, healthcare provider's influence on the decision on venue of delivery, possession of valid health insurance card, and SES (Table 6). In the multivariable analyses, site of residence, healthcare provider's influence on the decision on venue of delivery, possession of valid health insurance card, and SES were associated with place of delivery. The women living in Navrongo were more likely to deliver at health facilities than women living in Dodowa [adjusted odds ratio (AOR) = 4.49; 95% CI (1.14–17.68)]. There was no statistically significant difference in health facility delivery between Kintampo and Dodowa.

**Table 6:** Determinants of health facility delivery across all three sites.

Characteristics	Crude OR	(95% CI)	Adjusted OR	(95% CI)
**Age of mothers (N = 1,480)**				
10–19 years	1			
20–29 years	0.97	(0.62–1.50)		
30–39 years	0.88	(0.56–1.38)		
40–49 years	1.09	(0.58–2.04)		
**Age of partners (N = 1,049)**				
< 29	1			
30–39	1.1	(0.77–1.59)		
40–49	0.96	(0.62–1.49)		
> 50	1.05	(0.53–2.10)		
**Marital status (N = 1,497)**				
Married	1		1	
Cohabiting	0.59	(0.45–0.77)***	0.69	(0.38–1.28)
Divorced/separated/widowed	0.89	(0.45–1.79)	0.87	(0.30–2.55)
Never married	0.67	(0.45–0.99)*	0.63	(0.30–1.31)
**Ethnicity (N = 1,422)**				
Northern tribes	1		1	
Akan	0.7	(0.52–0.94)*	0.64	(0.19–2.21)
Ga/Adangbe/Ewe	1.32	(0.90–1.95)	1.79	(0.77–4.13)
Others	0.48	(0.34–0.69)***	0.97	(0.50–1.85)
**Religion (N = 1,426)**				
Christian	1		1	
Islam	0.55	(0.40–0.76)***	0.85	(0.43–1.68)
Traditional	1.39	(1.02–1.90)*	0.58	(0.25–1.34)
Other	1.1	(0.63–1.93)	2.12	(0.77–5.88)
**Educational attainment of mother (N = 1,497)**				
None	1		1	
Primary	1.46	(1.09–1.96)*	0.83	(0.49–1.39)
Middle/JSS/JHS	2.71	(1.98–3.69)***	1.68	(0.96–2.95)
Secondary/SSS/SHS/Tech/Voc	6.66	(3.17–13.94)***	1.85	(0.66–5.21)
Tertiary and above	17.06	(2.32–125.63)**	1.36	(0.15–12.45)
**Educational attainment of partner (N = 1,370)**				
None	1		1	
Primary	1.28	(0.88–1.86)	0.61	(0.33–1.12)
Middle/JSS/JHS	1.73	(1.27–2.35)***	1.14	(0.66–1.97)
Secondary/SSS/SHS/Tech/Voc	2.91	(1.90–4.45)***	0.92	(0.46–1.82)
Tertiary and above	5.86	(2.77–12.38)***	0.85	(0.30–2.38)
**Number of births at last delivery (N = 1,497)**				
≤ 4 children	1		1	
> 4 children	0.72	(0.56–0.92)**	1.04	(0.66–1.63)
**ANC attendance (N = 1,497)**				
< 4 times	1		1	
≥ 4 times	3.31	(2.45–4.49)***	1.67	(0.94–2.95)
**Desire for pregnancy (N = 1,497)**				
Wanted at time of conception	1			
Wanted later	0.79	(0.61–1.03)		
Did not want at all	0.73	(0.49–1.09)		
**Education on danger signs of pregnancy during ANC (N = 1,472)**				
No	1		1	
Yes	1.45	(1.12–1.89)**	0.97	(0.62–1.51)
**Influence on woman’s decision on delivery venue (N = 1,496)**				
Non-healthcare provider	1		1	
Healthcare provider	21.52	(10.56–43.85)***	13.47	(5.96–30.48)***
**Possess a valid health insurance card (N = 1,091)**				
No	1		1	
Yes	2.17	(1.61–2.93)***	1.9	(1.29–2.81)**
**Wealth quintiles (N = 1,497)**				
Least wealthy	1		1	
Less wealthy	0.75	(0.53–1.05)	1.03	(0.58–1.85)
Wealthy	0.59	(0.42–0.84)**	1.24	(0.67–2.31)
Wealthier	1.21	(0.83–1.76)***	2.83	(1.43–5.60)**
Wealthiest	4.77	(2.83–8.03)***	6.81	(2.99–15.50)***
**Site of residence (N = 1,497)**				
Dodowa	1		1	
Kintampo	0.51	(0.39–0.67)***	0.41	(0.15–1.15)
Navrongo	2.68	(1.88–3.80)***	4.49	(1.14–17.68)*

Covariates which were significant in multivariable logistic analyses were included:

* p < .05

** p < .005

*** p < .001

Women who were influenced on the decision regarding venue of delivery by a healthcare provider were more likely to deliver at a health facility than were those who were influenced by a non-healthcare provider [AOR = 13.47; 95% CI (5.96–30.48)]. Women with a valid health insurance card were more likely to deliver in a health facility as compared to those without [AOR = 1.90; 95% CI (1.29–2.81)]. Compared to the least wealthy, the wealthiest [AOR = 6.81; 95% CI (2.99–15.50)] and wealthier [AOR = 2.83; 95% CI (1.43–5.60)] women were 7 and 3 times more likely to deliver in health facilities respectively.

### Determinants of health facility delivery at individual sites

Regression analyses for the individual sites were performed with those same variables used across all the sites (Table 7), with the exception of site of residence. Healthcare provider’s influence on women's decision on delivery venue had a positive association with health facility delivery in Dodowa [AOR = 61.19; 95% CI (6.89–543.22)], Kintampo [AOR = 10.53; 95% CI (3.38–32.77)], and Navrongo [AOR = 7.46; 95% CI (1.67–33.30)] (Table 7). In Dodowa, those with valid insurance cards were more likely to deliver within health facilities than were women without valid health insurance, [AOR = 3.14; 95% CI (1.30–7.56)]. In Kintampo, the wealthiest [AOR = 16.00 95% CI (3.81–67.17)] women were more likely to deliver at health facilities than the least wealthiest.

**Table 7:** Determinants of health facility delivery at individual study sites.

Characteristics	Dodowa (n = 500)		Kintampo (n = 500)		Navrongo (n = 497)	
	AOR	(95% CI)	AOR	(95% CI)	AOR	(95% CI)
**Influence on woman’s decision on delivery venue**						
Non-healthcare provider	1		1		1	1
Healthcare provider	61.19	(6.89–543.22)***	10.53	(3.38–32.77)***	7.46	(1.67–33.30)**
**Possess a valid health insurance card**						
No	1		1		1	1
Yes	3.14	(1.30–7.56)*	1.72	(0.95–3.09)	1.81	(0.81–4.07)
**Wealth quintiles**						
Least wealthy	1		1		1	1
Less wealthy	0.38	(0.05–2.64)	0.79	(0.28–2.22)	1.60	(0.89–3.75)
Wealthy	1.03	(0.21–5.00)	0.76	(0.29–2.03)	3.01	(0.72–12.71)
Wealthier	3.27	(0.66–16.17)	2.38	(0.84–6.75)	0.00	(0.00–0.00)
Wealthiest	2.57	(0.51–12.92)	16.00	(3.81–67.17)***	3.16	(0.33–30.40)

* p < .05

**p < .005

*** p < .001

## Discussion

The study determined the factors that contribute to delivery in health facilities in predominantly rural communities in Ghana. The study found that healthcare provider’s influence on the delivery venue decision, possession of valid health insurance card, higher socio-economic status and living in Navrongo were associated with health facility delivery.

Women delivered more at health facilities across all three sites when healthcare providers influenced the women’s decision for health facility delivery. This suggests that counseling pregnant women on the importance of health facility delivery could facilitate health facility delivery. The influence of partners, relatives, friends, and mothers themselves on venue of delivery has been documented in previous studies [10, 11, 39]. However, literature is limited on the effects of healthcare provider counseling on health facility delivery. A recent study in Ethiopia highlighted its importance in enhancing health facility delivery for all pregnant women [40]. Also, healthcare providers were more likely to insist on health facility delivery for women with identified risks than those with normal pregnancies. This led to women with normal pregnancies delivering at home in other settings [15, 41]. Messages from healthcare providers should therefore be packaged to convey succinct information on the importance of health facility delivery. Further, as observed in the study, high ANC attendance [5, 42] provides an opportunity for healthcare providers to educate on the benefits of health facility delivery to pregnant women.

The proportion of health facility delivery across the three sites was slightly higher as compared to previous Ghanaian studies [23], and comparable to other communities in Ethiopia and Namibia [21, 43]. This study showed that about 25% of women gave birth outside of health facilities, which indicates that these women and their newborns were at risk of morbidity and mortality. Among the three sites in the study, the proportion of health facility delivery was the highest in Navrongo. A potential explanation is that, the CHPS concept in Ghana started in Navrongo as a research program “the Navrongo Experiment” from 1994 to 1997 [44, 45]. Navrongo has been at the forefront of the CHPS concept. It has built on the benefits of earlier primary healthcare programs by including components of MNCH [46–49]. Further, most CHOs in Navrongo have midwifery training [31], which allows women with no complications to deliver at CHPS compounds. A well-established CHPS concept might facilitate equal opportunity for delivery services to women in Navrongo.

Having valid health insurance was associated with health facility delivery across all study sites, particularly in Dodowa. This is in line with previous findings from other African countries where insurance-based programs and fee exemptions result in higher rates of health facility delivery [21, 22]. In Ghana, persons with health insurance receive healthcare services, including MNCH services, without out-of-pocket payments. Pregnant women with insurance are therefore more likely to opt for health facility delivery [50]. However, there are several challenges that affect poor uptake of health insurance in Ghana. Local interpretations of health insurance benefits have differed and served as a barrier to MNCH service utilization in Ghana [51]. Some services were not covered by health insurance packages, which compels clients to make out-of-pocket payments [51]. Furthermore, for the benefits of insurance to be felt, services would have to be physically accessible in the first place [51]. A well-established CHPS could provide some explanation as to why health facility delivery in Navrongo was not influenced by health insurance. Meanwhile, in Kintampo, women had limited access to delivery care in their communities, even though they may possess health insurance cards. Dodowa is located about a one-hour drive away from the capital of Accra. Such geographic positioning provides more options of delivery place to women. However, we do not have sufficient information to explain the association between possession of a health insurance card and health facility delivery in Dodowa. It will be worth conducting further investigations.

Wealthier women delivered more at health facilities than poorer ones across all three sites, particularly in Kintampo. The findings were similar in other resource-limited settings [9, 10, 20, 21, 52, 53]. The differences in the influence of socio-economic status on health facility delivery among the three study sites could be attributed to the level of development of the CHPS program. The program is much more established in Navrongo and Dodowa [31], and most CHOs have midwifery skills in the two sites. Cost of care could also be a reason for the relatively lower proportion of health facility deliveries in Kintampo [54], as women need to go outside their communities for health facility delivery. The cost for transportation might be a crucial barrier to accessing health facilities for women of lower economic status in Kintampo.

### Limitations

This study had several limitations. First, information used in this study was based on respondents’ voluntary answers. Therefore, there is a possibility of recall bias. To minimize this, we cross-checked the data on antenatal and delivery histories with the maternal health record book during the interviews. Second, this is a cross-sectional study, so we cannot define causality. We therefore described associations between outcome and explanatory variables. Despite these limitations, we captured the geographical diversity of the population by sampling from the southern, central and northern belts of Ghana, making it representative of the population.

## Conclusion

In addition to known factors such as place of residence, socio-economic status, and possession of valid health insurance, this study identified one more factor associated with health facility delivery: healthcare provider’s influence on women’s delivery venue decision.

The Ghana Health Service/Ministry of Health should institute policies that enable and ensure that healthcare providers counsel all pregnant women on preparing for health facility delivery and its benefits to their health and that of their neonates.

## Supporting Information

S1 File Questionnaire. (PDF)

## Abbreviations

CHPS: Community-Based Health Planning and Service
EMBRACE: Ensure Mothers and Babies Regular Access to Care
GHS: Ghana Health Service
HDSS: Health and Demographic Surveillance System
HRC: Health Research Centre
CHO: Community Health Officer
