# Supplementary material for: Factors Influencing Health Facility Delivery in Predominantly Rural Communities across the Three Ecological Zones in Ghana: A Cross-Sectional Study
Source: PLoS One. 2016 Mar 31;11(3):e0152235. doi: 10.1371/journal.pone.0152235 (PMC4816577; doi:10.1371/journal.pone.0152235)
Supplement: S1 File — (PDF) [file pone.0152235.s001.pdf]

S1File. Questionnaire

Time that this interview begins: | | | : | | |

Questionnaire: CoC Situation Analysis

KINTAMPO HEALTH RESEARCH CENTRE<br>EMBRACE FORMATIVE RESEARCH<br>FR2 FORM (25/07/2013)	FR2 FORM NO	FORMNO

PART 1: BASIC INFORMATION

1.1 Name of interviewer:		INAME
1.2 Staff code		FW
1.3 Date of interview **(DD/MM/YY)**:		DINT
1.4 Community Name		COMNAME
1.5 Compound Number		COMPNO
1.6 Mother's Perm ID		MID
1.7 Mother's Name		MNAME

NB: For the PERMID, please use the KHDSS card

Part 2: Background Information

2.1 Age of the mother (in completed years)

[ ][ ] Years old — PAR

2.2 Education level that the mother completed

1. None	2. Primary	3.Middle/JSS/JHS	4. Secondary/SSS/SHS/TECH/VOC	EDU
5. Tertiary or above		8. DK		

2.3 Number of pregnancy/Pregnancies (at the time of the last delivery)

[ ][ ] Times — IMAGE

2.4 What was the outcome of your last pregnancy?

★If your infant did not cry, move, or breathe when it was born, choose "Still birth".

1. Born and lived for 6 weeks or more	2. Born alive and died within 6 weeks	3. Still birth	TR
4. Miscarriage	8.Abortion		

2.5 Date of the last delivery or Date that your last pregnancy ended (**DD/MM/YY**)

[ ][ ][ ][ ][ ][ ] — DDAT

2.6 Age of your infant at the date of interview (month completed)

★If your infant is aged less than 1 month, fill out"00".

★If the outcome of the pregnancy is 4. "Miscarriage" or 5 "Abortion" in 2.4, fill out 99

[ ][ ] Months — PAR

2.7 Current marital status

★If "2. Cohabiting", "3. Divorced", "4. Separated" or "5. Never married", skip to 2.9.

1. Married	2. Cohabiting/ Living together	3. Divorced	4. Separated	5. Widowed	6. Never married	MARSTU

2.8 **<u>If "1. Married" in 2.7</u>**, how many years have you been married (years completed)?

[ ][ ][ ] Years — MARYR

2.9 Education level of partner or husband

1. None	2. Primary	3.Middle/JSS/JHS	4. Secondary/SSS/SHS/TECH/VOC	PEDU
5. Tertiary or above		8. DK		

2.10 Age of partner or husband (in completed years)

[ ][ ][ ] Years old — PAGE

2.11 At the time you became pregnant, did you want to become pregnant <u>then</u>, did you want to wait until <u>later</u>, or did you <u>not want</u> to have any (more) children at all?

1. Then	2. Later	3. Did not want at all	MPRE

Part 3: Antenatal Care (ANC)

◆<u>ANC</u> I would like to ask about antenatal care that you received in the last pregnancy.

3.1 How many times did you receive ANC during the last pregnancy?

1. None	2. 1 time	3. 2 times	4. 3 times	5. 4 times or more	6. DK but at least more than 1 time	8. DK	ANC

★If "1. None" or "8. DK", <u>skip to 3.5.</u>

3.2 What made you decide to receive ANC for the first time?

★Choose one.

1. Just wanted to get a check-up	2. Had (a) health problem(s)	RANC
3. Worries about pregnancy/delivery	4. Suggested by family/friend	
5. Suggested by health workers		
6. Other ( )		

3.3.1 Were you satisfied (happy) with the services provided at the ANC?

★If "1. Yes", skip to 3.3.3

1. Yes	2. No	8. DK	SANC

3.3.2 **If "2. No" in 3.3.1**, what were you **un**satisfied (unhappy) with at ANC?

★Multiple answers

1. Health care received from health workers	2. Long waiting	3. Neatness of health facility	UNANC
4. Emotional care or counselling received from health workers		5. Attitude of health workers	
6. Others ( )			

3.3.3 Where did you go for the ANC?

11. Public hospital/Polyclinic	12. Private hospital	13. Health centre		P_ATPC
14. Private clinic/maternity home	15. CHO office/CHPS/Community clinic	16. TBA's home		
17. Outreach clinic	18. Home	19.Other ( )	99. DK	

3.3.4 How did you get to the place you had the ANC?

★Choose the most typical mode of transportation. In case of receiving care at home, choose NA.

11. On foot	12. Bicycle	13. Tricycle/motor king	14. Motorcycle	15. Taxi	TR_ATPC
16. Public transport	17. Private car	18. Ambulance	99. DK	88. NA	

3.3.5How long did it take to go to the place for the ANC?

★Answer by minutes. ★Fill in "000" in case of receiving care at home.

			minutes	M_ATPC

3.4 Did you receive the following knowledge or care at ANC?

★Ask one by one.★After answering the question, skip to 3.6.

3.4.1. Danger signs of pregnancy	1. Yes	2. No	8. DK	DNANC
3.4.2. Family planning	1. Yes	2. No	8. DK	FPANC
3.4.3. Tetanus toxoid immunization (TT)	1. Yes	2. No	8. DK	TXANC
3.4.4. Nutrition	1. Yes	2. No	8. DK	NUTANC
3.4.5. Malaria (Intermittent Preventive Treatment)	1. Yes	2. No	8. DK	IPTANC

3.5 **If the answer was "None" in 3.1**, why were you not able to go for ANC during the last pregnancy?

★Ask as an open-ended question first. Then ask about each of the barriers listed below.

3.5.1. Not necessary to receive ANC	1. Yes w/o prompt	2. Yes on prompt	3. No	WOANC_NN
3.5.2. Not customary to receive ANC	1. Yes w/o prompt	2. Yes on prompt	3. No	WOANC_NC
3.5.3. Too early pregnancy stage to receive ANC (will go later)	1. Yes w/o prompt	2. Yes on prompt	3. No	WOANC_TE
3.5.4. Want to hide pregnancy	1. Yes w/o prompt	2. Yes on prompt	3. No	WOANC_WH
3.5.5. Shy to go to ANC	1. Yes w/o prompt	2. Yes on prompt	3. No	WOANC_SH
3.5.6. Religious reason	1. Yes w/o prompt	2. Yes on prompt	3. No	WOANC_RL
3.5.7. Transportation is not available or far distant	1. Yes w/o prompt	2. Yes on prompt	3. No	WOANC_TR
3.5.8. No one to accompany with	1. Yes w/o prompt	2. Yes on prompt	3. No	WOANC_AC
3.5.9. Cannot afford	1. Yes w/o prompt	2. Yes on prompt	3. No	WOANC_CA
3.5.10. Other ( )				WOANC_OT

◆ HIV/AIDS

3.6 During any of the antenatal visits for your last birth, did anyone talk to you about the following?

2.6.1.Babies getting HIV/AIDS from their mother	1. Yes	2. No	8. DK	HIV_MTCT
2.6.2.Thingsyou can do to prevent you from getting HIV/AIDS	1. Yes	2. No	8. DK	HIV_T
2.6.3.Getting tested for HIV/AIDS	1. Yes	2. No	8. DK	HIV_TEST

3.7 Did you take an HIV test at ANC? **You do not have to tell me the test result!**

1. Yes	2. No	8. DK	PMTCT_IF

◆Health Complications

3.6.1Did you have complications during the last pregnancy? (Subjective symptoms):

★*If "2. No" or "8. DK", skip to Part 4*

1. Yes	2. No	8. DK	CANC

3.6.2 **If "1. Yes" in 3.6.1**, (a) what were the complications (list up to 5 complications from the list below and others not in the list)?

(b) For each of the complications, who provided care?

★***Ask as an open-ended question first. Then ask about each complication based on the codes below.***

	a. Complication			b. Care provider	
1. Complication 1			CANCA		CANCPA
2. Complication 2			CANCB		CANCPB
3. Complication 3			CANCC		CANCPC
4. Complication 4			CANCD		CANCPD
5. Complication 5			CANCE		CANCPE
6. Complications not listed below			CANCF		CANCPF

Code of complications (a)

11. Vaginal bleeding<br>12. Abdominal pain<br>13. Swelling feet	14. Hyperemesis (Severe vomiting in pregnancy)<br>15. Unusual vaginal discharge<br>16. Severe headache	17. Fever<br>18. Severe anaemia<br>19. Dizziness<br>99. NA

Code of Care providers (b)

11. Did not seek care<br>12. Public hospital/Polyclinic<br>13. Private hospital<br>14. Health centre<br>15. Private clinic/maternity home	16. CHO office/CHPS/Community clinic<br>17. TBA's home<br>18. Outreach clinic<br>19. Herbalist/Spiritualist<br>20. Chemical seller/Pharmacist	21. Prayer camp<br>22. Other<br>88. DK<br>99. NA

Part 4: Delivery Care

◆Place and Birth Assistant I would like to ask about the place and the assistant for your last delivery.

4.1 Place of delivery:

11. Public hospital/Polyclinic	12. Private hospital	13. Health centre	PDL
14. Private clinic/maternity home	15. CHO office/CHPS/Community clinic	16. TBA's home	
17. Outreach clinic	18. On the way to facility	19. Home	
20. Other ( )		88. DK	

4.2. Who decided the place for delivery?

★***Multiple answer.***

11. No one	12. Doctor/Nurse/midwife/CHN/CHO/EN		13. Traditional birth attendant	DMDL
14. Husband	15. Mother	16. Mother-in-law	17. Relative/friend	
18. Other ( )			88. DK	

4.3.1 Who assisted you during delivery?

11. No one	12. Doctor/Nurse/midwife/CHN/CHO/EN		13. Traditional birth attendant	ATDL
14. Husband	15. Mother	16. Mother-in-law	17. Relative/friend	
18. Other ( )			88. DK	

★***If the answer is other than "11. Self." in 4.3.1, <u>skip to 4.4.</u>***

4.3.2. **<u>If the answer was "No one" in 4.3.1</u>,** what is the reason you did not get assistance during the last delivery?

★***Ask as an open-ended question first. Then ask about each of the barriers listed below.***

4.3.2.1. Feel shy	1. Yes w/o prompt	2. Yes on prompt	3. No	NATDL_AS
4.3.2.2. Don't want to waste other's time	1. Yes w/o prompt	2. Yes on prompt	3. No	NATDL_NW
4.3.2.3. My husband did not allow	1. Yes w/o prompt	2. Yes on prompt	3. No	NATDL_HN
4.3.2.4. My mother-in-law didn't allow	1. Yes w/o prompt	2. Yes on prompt	3. No	NATDL_MON
4.3.2.5. Lack of phone/communication tools	1. Yes w/o prompt	2. Yes on prompt	3. No	NATDL_NA
4.3.2.6. Lack of money	1. Yes w/o prompt	2. Yes on prompt	3. No	NATDL_LMN
4.3.2.7. No facility nearby	1. Yes w/o prompt	2. Yes on prompt	3. No	NATDL_NF
4.3.2.8.Unprompted or accidentally	1. Yes w/o prompt	2. Yes on prompt	3. No	NATDL_UP
4.3.2.9. Always delivered by myself	1. Yes w/o prompt	2. Yes on prompt	3. No	NATDL_DM
4.3.2.10. Other ( )				NATDL_OT

4.4 Who decided the on the person to assist you during delivery?

★***Multiple answer***

11. No one	12. Doctor/Nurse/midwife/CHN/CHO/EN		13. Traditional birth attendant	DATDL
14. Husband	15. Mother	16. Mother-in-law	17. Relative/friend	
18. Other ( )			88. DK	

4.5.1Birth weight (kg) of your infant (from the health card)

★***If mother answers this question, skip to 4.6.1.***

[ ] . [ ] Kg (from the health card) BTWTR

4.5.2**(If 4.5.1 is not available)** Birth weight (kg) of your infant (based on mother's recall)

[ ] . [ ] Kg (from the health card) BTWTM

4.5.3What did you think about the size of your infant immediately after the delivery?

1. Very small	2. Small	3. Average	4. Large	5. Larger than usual	BTWPC

◆ <u>Health Complications</u>

4.6.1 Did you have complications during the last delivery?

★***If "2. No" or "8. DK", skip to 4.7***

1. Yes	2. No	8. DK	CDL

4.6.2**If "Yes" in 4.6.1.**, what kind of complications did you have during the last delivery (up to 5 complications listed below and other complications not in the list)?

★***Ask as an open-ended question first. Then ask about each complication listed below.***

	a. Complication		
1. Complication 1			CDLCA
2. Complication 2			CDLCB
3. Complication 3			CDLCC
4. Complication 4			CDLCD
5. Complication 5			CDLCE
6. Complications not listed below			CDLCOT

Code of complications

1. Heavy bleeding	6. Multiple-birth
2. Preterm birth	7. Placenta not expelled 1 hour after birth of the baby
3. Premature rupture of the membrane (water break and not in labour after 6 hours)	8. Fever
4. Prolonged delivery (Labour pains/contractions continue for more than 12 hours)	9. Stillbirth
5. Breech, foot, or compound presentation	10. Convulsion
	11. Vesicovaginal fistula (VVF)
	12. Rupture uterus

4.7Did your infant have any of the following danger signs immediately after the delivery?

★*Ask one by one*

4.7.1. Was he/she very small	1. Yes	2. No	8. DK	CN_VS
4.7.2. Had difficulty in breathing	1. Yes	2. No	8. DK	CN_BR
4.7.3. Had fever	1. Yes	2. No	8. DK	CN_FV
4.7.4. Cold body	1. Yes	2. No	8. DK	CN_CO
4.7.5. Bleeding	1. Yes	2. No	8. DK	CN_BL
4.7.6. Too weak to suck/feed	1. Yes	2. No	8. DK	CN_NF
4.7.7. Did not cry	1. Yes	2. No	8. DK	CN_NC

◆Home Delivery If you delivered at home or at TBA's home, please answer the following questions.

★***Check the question in 4.1. If delivered at facility, skip to 4.11.1.***

4.8.1Did the attendant wash her hands before attending to you?

1. No attendant	2. Yes, with soap and water	3. Yes, with water only	4. No	8. DK	HD_WH

4.8.2 Did you deliver on a clean bed / on a floor covered with clean sheet?

1. Yes	2. No	8. DK	HD_CS

4.9Did you have a vehicle ready to take you to a health facility if complications occur?

1. Yes	2. No	8. DK	HD_G

4.10.1Were you satisfied (happy) with the care received during the delivery?

★*If "2. No", "3. No attendant" or "8. DK", skip to Part 5*

1. Yes	2. No	3. No attendant	8. DK	SHD

4.10.2 **If "2. No" in 4.10.1**, what were you unsatisfied (unhappy) with during the delivery?

★*Multiple answers*

1. Medical care by attendants	2. Emotional care or counselling by attendants	USHD
3. Attitude of attendants	4. Others ( )	

◆Facility Delivery If you delivered at health facility, please answer the following questions.

4.11.1How did you get to the health facility?

11. On foot	12. Bicycle	13. Tricycle/motor king	14. Motorcycle	15. Taxi	TR_FDL
16. Public transport	17. Private car	18. Ambulance	8. DK		

4.11.2 How long did it take you to get to the place of delivery (Facility)?

			minutes	M_FDL

4.12.1Were you satisfied (happy) with the care received during the delivery?

1. Yes	2. No	3. No attendant	8. DK	SFDL

4.12.2 **If "2. No" in 4.12.1**, what were you unsatisfied (unhappy) with during the delivery?

★*Multiple answers*

1. Medical care by attendants	2. Emotional care or counselling by attendants	USFDL
3. Attitude of attendants	4. Others ( )	

4.13. Did you receive instructions (or were you told) on when to come for postnatal check-up?

1. Yes	2. No	8. DK	ICDL

4.14. Who stayed with you at health facility for the first 24 hours after delivery?

★Multiple answers

11. No one	12. Doctor/Nurse/midwife/CHN/CHO/EN		13. Traditional birth attendant	ACDL
14. Husband	15. Mother	16. Mother-in-law	17. Relative/friend	
18. Other ( )			88. DK	

◆Neonatal Care **I would like to ask about care that your infant received just after birth.**

4.15.1 Was your infant dried soon after birth?

1. Yes	2. No	8. DK	DNDL

4.15.2 Was your infant put on your chest (skin-to-skin care) after the health worker finished working on him/her?

1. Yes	2. No	8. DK	SNDL

4.15.3 Was new (disposable) ties and razor blade used to cut and tie the cord?

1. Yes	2. No	8. DK	CNDL

Part 5: Postnatal Care (PNC) of Mother

◆PNC **I would like to ask about postnatal care that you received in the last delivery.**

5.1 Did you receive **your (maternal)** health check-up after the delivery?

★If answer is "2. No" or "8. DK", skip to 5.10.1.

1. Yes	2. No	8. DK	MPC

5.2 When was the first check-up after delivery?

★Fill out "00" if they visit it at the same day of the delivery.

		days after delivery	FMPC

5.3 Who checked on **your health** at the first PNC?

1. Doctor/Nurse/Midwife/CHN/CHO/EN	2. Traditional birth attendant		WOMPC
3. Other ( )		8. DK	

5.4 Where did you receive the first PNC?

11. Public hospital/Polyclinic		12. Private hospital	13. Health centre	PMPC
14. Private clinic/maternity home		15. CHO office/CHPS/Community clinic	16. TBA's home	
		19. Other ( )	88. DK	

5.5 How did you get to the place for the first PNC?

★Choose the most typical mode of transportation. In case of receiving care at home, choose NA.

11. On foot	12. Bicycle	13. Tricycle/motor king	14. Motorcycle	15. Taxi	TRPNC
16. Public transport	17. Private car	18. Ambulance	88. DK	99. NA	

5.6 How long did it take you to get to the place of PNC?

			minutes	MMPC

5.7 What made you decide to receive the PNC?

★Choose one

1. Just wanted to get a check-up	2. Had health problem(s)	3. Worries about delivery	RMPC
4. Suggested by family/friends	5. Suggested by health workers beforehand		
6. Other ( )			

5.8 Did you receive the following knowledge or care at PNC?

★*Ask one by one.*

5.8.1. Complications of mother and infants	1. Yes	2. No	8. DK	KMPCM
5.8.2 Self care of mother and infants	1. Yes	2. No	8. DK	KMPCSC
5.8.3. Family planning	1. Yes	2. No	8. DK	KMPCFP
5.8.4. Nutrition and anaemia	1. Yes	2. No	8. DK	KMPCN
5.8.5. Breastfeeding	1. Yes	2. No	8. DK	KMPCBF
5.8.6. Immunization	1. Yes	2. No	8. DK	KMPCI

5.9 Did you receive home visit by health workers within 6 weeks after delivery?

1. Yes	2. No	8. DK	MPCHV

◆Health Complications

5.10.1Did you have any complications within 6 weeks after delivery?

★***If answer is "2. No" or "8. DK", skip to Part 6.***

1. Yes	2. No	8. DK	CMPC

5.10.2 **If "1. Yes" in 5.10.1**, (a) what were the complications (list up to 5 complications)?

(b) For each of the complications, who provided care?

★***Ask as an open-ended question first. Then ask about each complication based on the codes below.***

	a. Complication			b. Care provider	
1. Complication 1			CMPCA		CMPCPA
2. Complication 2			CMPCB		CMPCPB
3. Complication 3			CMPCC		CMPCPC
4. Complication 4			CMPCD		CMPCPD
5. Complication 5			CMPCE		CMPCPE
6. Complications not listed below			CMPCF		CMPCPF

Code of complications (a)

11. Perinea swelling<br>12. Problem in urination<br>13. Fever/Chills<br>14. Breast problem (engorgement, pain in nipple or breast)<br>15. Easily felt tired<br>16. Felt breathless during routine household work	17. Felt unhappy or crying easily<br>18. Severe bleeding<br>19. Convulsion<br>20. Foul smelling or purulent vaginal discharge<br>21. Passing urines through vagina (fistula)<br>99. NA

Code of Care providers (b)

11. Did not seek care<br>12. Public hospital/Polyclinic<br>13. Private hospital<br>14. Health centre<br>15. Private clinic/maternity home	16. CHO office/CHPS/Community clinic<br>17. TBA's home<br>18. Outreach clinic<br>19. Herbalist/Spiritualist<br>20. Chemical seller/Pharmacist	21. Prayer camp<br>22. Other<br>88. DK<br>99. NA

Part 6. Infant PNC and Weighing

◆ Feeding Practice for the last child **I would like to ask about the feeding practices of your last child.**

★ ***If the answer of 2.4 is "still birth", then skip to Part 7 and cross out the questions.***

6.1.1When did you initiate breastfeeding after delivery?

1.Within one hour	2. After one hour but within 24 hours	3. After 24 hours	8. DK/Don't remember	INBF

6.1.2 Have you ever given anything other than breast milk to your infant (**before 6 months of age)**?

1. Yes	2. No	8. DK	EBF

★ *If the answer is "2. No" or "8. DK", skip to 6.2.*

6.1.3**If "Yes" in 5.1.2,** what did you give to your infant other than breast milk **before 6 months of age**?

★***Ask as an open-ended question first. Then ask about each of the items listed below.***

6.1.3.1. Untreated/Wild water (from river, stream etc)	1.Yes	2. No	8. DK	IF_W
6.1.3.2. Boiled water	1.Yes	2. No	8. DK	IF_BE
6.1.3.3. Bottled water/sachet water	1.Yes	2. No	8. DK	IF_BT
6.1.3.4. Tea	1.Yes	2. No	8. DK	IF_T
6.1.3.5. Juice/soup	1.Yes	2. No	8. DK	IF_J
6.1.3.6. Weaning food (specially prepared for the baby)	1.Yes	2. No	8. DK	IF_WF
6.1.3.7. Solid food (part of family food)	1.Yes	2. No	8. DK	IF_SF
6.1.3.8. Infant formula	1.Yes	2. No	8. DK	IF_F
6.1.3.9. Other ( )				IF_OT

6.2. Did you take your infant to postnatal care, child welfare clinic (CWC) or weighing?

1. Yes	2. No	8. DK	IPC

★ *If the answer is "No", skip to 6.7 (about immunization).*

◆Infant check-up (weighing) at 2 days postnatal

6.3.1 Did you take your infant to the check-up **within 2 days after delivery**?

1. Yes	2. No	8. DK	DTIPC

★*If answer is "2. No" or "8. DK", skip to 6.4.1.*

6.3.2 When was the first visit after delivery?

★***Fill out "00" if the visit is on the same day of the delivery.***

[ ][ ] days after delivery D_DTPC

6.3.3 Where did you take your infant for the check-up **within 2 days after delivery**?

11. Public hospital/Polyclinic	12. Private hospital	13. Health centre	P_DTPC
14. Private clinic/maternity home	15. CHO office/CHPS/Community clinic	16. TBA's home	
17. Outreach clinic / 18. Home	19. Other ( )	88. DK	

6.3.4 How did you get to the place for the check-up **within 2 days after delivery**?

★***Choose the most typical mode of transportation. In case of receiving care at home or in the community, choose NA.***

11. On foot	12. Bicycle	13. Tricycle/motor king	14. Motorcycle	15. Taxi	TR_DTPC
16. Public transport	17. Private car	18. Ambulance	88. DK	99. NA	

6.3.5 How long did it take you to go for the check-up within 2 days after delivery?

★***Answer by "minutes".*** ★***Fill in "0" when the infant received PNC at home/in the community.***

[ ][ ][ ] minutes M_DTPC

6.3.6 What made you decide to take your infant for check-up **within 2 days after delivery**?

★***Choose one.***

1. Just wanted to get a check-up	2. Had (a) health problem(s)	R_DTPC
3.Suggested by family / friends	4. Suggested by health workers beforehand	
5.Other ( )		

6.3.7 Who checked the health of your infant **within 2 days after delivery**?

1. Doctor/Nurse/Midwife/CHN/CHO/EN	2. Traditional birth attendant	WODPTC
3. Other ( )	8. DK	

◆Infant CWC at 2 weeks postnatal

6.4.1Did you take your infant to PNC or CWC **around 2 week after delivery**?

1. Yes	2. No	8. DK	WTPC

★If answer is "2. No" or "8. DK", skip to 6.5.1.

6.4.2 Where did you take your infant for PNC or CWC **around 2 weeks after delivery**?

11. Public hospital/Polyclinic		12. Private hospital	13. Health centre	P_WTPC
14. Private clinic/maternity home		15. CHO office/CHPS/Community clinic	16. TBA's home	
17. Outreach clinic	18. Home	19. Other ( )	88. DK	

6.4.3. How did you get to the place for Child Welfare Clinic (CWC) **around 2 weeks after delivery**?

★Choose the most major one. Choose NA in case the mother received care at home/in the community.

11. On foot	12. Bicycle	13. Tricycle/motor king	14. Motorcycle	15. Taxi	TRWTPC
16. Public transport	17. Private car	18. Ambulance	88. DK	99. NA	

6.4.4. How long did it take to go to PNC or CWC **around 2 weeks after delivery**?

★Answer by "minutes". ★Fill in "0" if the infant received PNC or CWC at home/in the community.

			minutes	M_WTPC

6.4.5 What made you decide to take your infant to PNC or CWC **around 2 weeks after delivery**?

★Choose one answer

1. Just wanted to get a check-up	2. Had (a) health problem(s)	R_WTPC
3 Suggested by family/friends	4. Suggested by health workers beforehand	
5.Others ( )		

6.4.6 Who checked the health of your infant at **around 2 weeks after delivery**?

1. Doctor/Nurse/Midwife/CHN/CHO/EN	2. Traditional birth attendant		WOWTPC
3. Other ( )		8. DK	

◆Infant CWC at 6 weeks postnatal

6.5.1 Did you take your infant to PNC or CWC **around 6 weeks after delivery**?

1. Yes	2. No	8. DK	WSPC

★If answer is "2. No" or "8. DK", skip to 6.6.

6.5.2Where did you take your infant for PNC or CWC **around 6 weeks after delivery**?

11. Public hospital/Polyclinic		12. Private hospital	13. Health centre	P_WSPC
14. Private clinic/maternity home		15. CHO office/CHPS/Community clinic	16. TBA's home	
17. Outreach clinic	18. Home	19. Other ( )	88. DK	

6.5.3How did you get to place for PNC or CWC **around 6 weeks after delivery**?

★Choose the most major one. Choose NA in case of received care at home.

11. On foot	12. Bicycle	13. Tricycle/motor king	14. Motorcycle	15. Taxi	TR_WSPC
16. Public transport	17. Private car	18. Ambulance	88. DK	99. NA	

6.5.4. How long did it take to go to PNC or CWC **around 6 weeks after delivery**?

★Answer by "minutes". ★Fill in "0" if the infant received PNC at home.

			minutes	M_ WSPC

6.5.5 What made you decide to take your infant to PNC or CWC at **around 6 weeks after delivery**?

★Choose one answer

1. Just wanted to get a check-up	2. Had (a) health problem(s)	R_WSPC
3.Suggestedby family/friends	4. Suggested by health workers beforehand	
5.Other ( )		

6.5.6 Who checked the health of your infant at **<u>around 6 weeks after delivery</u>**?

1. Doctor/Nurse/Midwife	2. Traditional birth attendant	3. CHN/CHO/EN	WO_ WSPC
4. Other ( )		8. DK	

◆ Infant Sickness within 6 weeks of age

6.7 Did your infant have any health problem **<u>within 6 weeks of age</u>**?

1. Yes	2. No	8. DK	IFH

★*If the answer is "2. No" or "8. DK", <u>skip to Part 7.</u>*

6.8 **If "1. Yes" in 6.7**, (a) what were the health problems (list up to 5 complications)?
(b) For each of the problems, how old was your infant when he or she was sick?
(c) When did you take your infant to a health facility at that time after recognizing the infant's sickness?
(d) Who provided care?

★***Ask as an open-ended question first. Then ask about each of health problems based on the codes below.***

	a. Health problem			b. How old(weeks)			c. When seeking care		d. Care provider	
1. Health problem 1			IFHCA			IFHWA		IFHRA		IFHCPA
2. Health problem 2			IFHCB			IFHWB		IFHRB		IFHCPB
3. Health problem 3			IFHCC			IFHWC		IFHRC		IFHCPC
4. Health problem 4			IFHCD			IFHWD		IFHRD		IFHCPD
5. Health problem 5			IFHCE			IFHWE		IFHRE		IFHCPE
6. Health problem not in the list below			IFHCF			IFHWF		IFHRF		IFHCPF

(a) Code of health problems

11. Refusal or inability to feed<br>12. Convulsed or fitted<br>13. Fever<br>14. Difficulties in breathing<br>15. Jaundice (severe)<br>16. Diarrhea<br>17. Sinking in the chest whilst breathing	18. Vomiting every feed<br>19. Lethargy or moving only when stimulated<br>20. Skin pustules or Boils<br>21. Excess crying<br>22. Cough<br>23. Not gaining weight<br>99. NA

(c) Code of "When seeking care after recognizing sickness"

1. Within a day<br>2. 1 to 3 days	3. 3 to 7 days<br>4. More than 7 days	8. DK<br>9. NA

(d) Code of Care providers

11. Did not seek care<br>12. Public hospital/Polyclinic<br>13. Private hospital<br>14. Health centre<br>15. Private clinic/maternity home	16. CHO office/CHPS/Community clinic<br>17. TBA's home<br>18. Outreach clinic<br>19. Herbalist/Spiritualist<br>20. Chemical seller/Pharmacist	21. Prayer camp<br>22. Other<br>88. DK<br>99. NA

Part 7. Health Seeking Behaviour

7.1.1 Do you have a health insurance card? (It should be valid as at the time of the interview)

1. Yes	2. No	8. DK	FHIC

★*If the answer is "2. No" or "8. DK", <u>skip to 7.2.1.</u>*

7.1.2 **If "Yes" in 7.1.1**, can you show me the health insurance card? (The card should be valid)

1.Yes, Valid	2. Yes, Not valid	2. No	8. DK	9. NA, no card	FHICS

7.2.1 Does your household have money readily available to seek care whenever necessary?

1. Yes	2. No	8. DK	FNHS

★*If answer is "2. No" or "8. DK", skip to 7.3.*

7.2.2 **If "Yes" in 7.2.1**, is that money readily available to you whenever you might need it (necessary)?

1. Yes	2. No	8. DK	FNHSF

7.3 Besides the financial support, does your family support you to visit health facilities/seek care for your own health whenever necessary?

★*Multiple answers*

1. Take care of your infant on behalf of you	2. Accompany them to health facilities	3. Encourage you to seek care	FSMHS
4. No other Support	5. Other (specify)		

7.4 Besides the financial support, do your family support you whenever you take your infant to the health facility when s/he is sick?

★*Multiple answers*

1. Take care of other children on behalf of you	2. Accompany them to health facilities	3. Encourage you to seek care	FSMHIS
4. No other Support	5. Other (specify)		

7.5 Do you receive physical, verbal or sexual violence from your husband/partner?

1. Yes, often	2. Yes, sometimes	3. No, not at all	DV

7.6 Do you believe that all infant illnesses can be treated?

1.Yes, all infant illnesses can be treated	2. Yes, some infant illnesses can be treated	3. No	8. DK	ITHF

◆Barriers to Access to Health Care

7.7 What kinds of difficulties made you reluctant to go to facilities? If any, when did the difficulty make you reluctant to go to facilities?

★*Ask as an open-ended question first. Then ask about each of the barriers listed below.*

	(a) Difficulty that made you reluctant to go to facility?				(b) If Yes in (a), when?	D_AFA
Health factor						
1. Felt tired / felt sleepy	1. Yes w/o prompt	2. Yes on prompt	3. No	D_FTA		D_FTB
2. Cannot walk	1. Yes w/o prompt	2. Yes on prompt	3. No	D_CWA		D_CWB
3. Blurred vision	1. Yes w/o prompt	2. Yes on prompt	3. No	D_BVA		D_BVB
4. Infant's illness (not severe)	1. Yes w/o prompt	2. Yes on prompt	3. No	D_IIA		D_IIB
5. Wait and see the symptom	1. Yes w/o prompt	2. Yes on prompt	3. No	D_WSA		D_WSB
Environmental/climate/ geographical factor						
6. Too far to go	1. Yes w/o prompt	2. Yes on prompt	3. No	D_FRA		D_FRB
7. Unable to go due to poor road network, distance, or transport	1. Yes w/o prompt	2. Yes on prompt	3. No	D_TRA		D_TRB
Family factor						
8. No one could accompany me to go to facility	1. Yes w/o prompt	2. Yes on prompt	3. No	D_ACA		D_ACB
9. Husband was not supportive about going to facility	1. Yes w/o prompt	2. Yes on prompt	3. No	D_HNSA		D_HNSB
10. Mother-in-law was not supportive about going to facility	1. Yes w/o prompt	2. Yes on prompt	3. No	D_MNSA		D_MNSB
11. Lack of money	1. Yes w/o prompt	2. Yes on prompt	3. No	D_MNA		D_MNB
12. Difficult to go along with other children	1. Yes w/o prompt	2. Yes on prompt	3. No	D_CHA		D_CHB
13. Infant is too young to travel	1. Yes w/o prompt	2. Yes on prompt	3. No	D_ITA		D_ITB
14. Religious reasons	1. Yes w/o prompt	2. Yes on prompt	3. No	D_RGA		D_RGB
Facility factor						

15. Did not know where to go	1. Yes w/o prompt	2. Yes on prompt	3. No	D_DNA		D_DNB
16. Poor quality of care	1. Yes w/o prompt	2. Yes on prompt	3. No	D_SRA		D_SRB
17. No doctor available	1. Yes w/o prompt	2. Yes on prompt	3. No	D_NFA		D_NFB
18. Inconvenient service hour	1. Yes w/o prompt	2. Yes on prompt	3. No	D_ISA		D_ISB
19. Went to facility, but turned away because it was too early to deliver	1. Yes w/o prompt	2. Yes on prompt	3. No			D_AFB
20. Long waiting time	1. Yes w/o prompt	2. Yes on prompt	3. No	D_LTA		D_LTB
21. Poor staff attitude	1. Yes w/o prompt	2. Yes on prompt	3. No	D_SCA		D_SCB
22. Facility closed when needed	1. Yes w/o prompt	2. Yes on prompt	3. No	D_FCA		D_FCB
23. Afraid to go	1. Yes w/o prompt	2. Yes on prompt	3. No	D_AGA		D_AGB
24. Sought for alternative treatment	1. Yes w/o prompt	2. Yes on prompt	3. No	D_ATA		D_ATB
25. Other	1. Yes w/o prompt	2. Yes on prompt	3. No	D_OTA		D_OTB

(b) Code of "When did the difficulty make you reluctant to go to facilities?"

1. Throughout pregnancy, delivery, and after delivery<br>2. During pregnancy and delivery<br>3. During pregnancy and after delivery<br>4. During delivery and after delivery	5. During pregnancy only<br>6. During delivery only<br>7. After delivery only<br>9. NA

◆Referral to Advanced Health / Medical Care

7.8 From the start of your pregnancy, through child birth, till 6 weeks after delivery, (a) were you referred to other health facilities for advanced medical care?

★If the answer is "No" in (a), end the interview here and cross out the rest of the form

	a. Referred or not			b. Went to that facility?			c. Which facility		
1. During pregnancy	1. Yes	2. No	RFSA	1. Yes	2. No	RFGA			RFFA
2. During delivery	1. Yes	2. No	RFSB	1. Yes	2. No	RFGB			RFFB
3. After delivery (within 6 weeks)	1. Yes	2. No	RFSC	1. Yes	2. No	RFGC			RFFC

(c) Code of "the Health facilities in the Study area where the woman is likely to be referred to"

Use the health facility key provided.	

7.9. Which of the following difficulties prevented you/could have prevented you from seeking advanced medical care?

★*Ask as an open-ended question first. Then ask about each of the barriers listed below.*

Health Factor				
1. Felt tired / felt sleep	1. Yes w/o prompt	2. Yes on prompt	3. No	RF_FT
2. Cannot walk	1. Yes w/o prompt	2. Yes on prompt	3. No	RF_CW
3. Blurred vision	1. Yes w/o prompt	2. Yes on prompt	3. No	RF_BV
4. Infant's illness (not severe)	1. Yes w/o prompt	2. Yes on prompt	3. No	RF_IL
5. Wait and see the symptom	1. Yes w/o prompt	2. Yes on prompt	3. No	RF_WS
Environmental/Climate/Geographical factor				
6. Too far to go to facilities	1. Yes w/o prompt	2. Yes on prompt	3. No	RF_FR
7. Unable to go to facilities due to poor road network, distance, or transport	1. Yes w/o prompt	2. Yes on prompt	3. No	RF_TR
Family factor				
8. No one could accompany me to go to facility	1. Yes w/o prompt	2. Yes on prompt	3. No	RF_AC
9. Husband was not supportive about going to facility	1. Yes w/o prompt	2. Yes on prompt	3. No	RF_HNS
10. Mother-in-law was not supportive about going to facility	1. Yes w/o prompt	2. Yes on prompt	3. No	RF_MNS
11. Lack of money	1. Yes w/o prompt	2. Yes on prompt	3. No	RF_MN
12. Difficult to go along with other children	1. Yes w/o prompt	2. Yes on prompt	3. No	RF_CH
13. Infant is too young to travel	1. Yes w/o prompt	2. Yes on prompt	3. No	RF_IT
14. Religious reasons	1. Yes w/o prompt	2. Yes on prompt	3. No	RF_RG
Facility factor				
15. Did not know where to go	1. Yes w/o prompt	2. Yes on prompt	3. No	RF_DN
16. Poor quality of care	1. Yes w/o prompt	2. Yes on prompt	3. No	RF_SR
17. No doctor available	1. Yes w/o prompt	2. Yes on prompt	3. No	RF_NF
18. Inconvenient service hour	1. Yes w/o prompt	2. Yes on prompt	3. No	RF_IS
19. Went to facility, but turned away because it was too early to deliver	1. Yes w/o prompt	2. Yes on prompt	3. No	RF_AF
20. Long waiting time	1. Yes w/o prompt	2. Yes on prompt	3. No	RF_LT
21. Poor staff attitude	1. Yes w/o prompt	2. Yes on prompt	3. No	RF_SC
22. Facility closed when needed	1. Yes w/o prompt	2. Yes on prompt	3. No	RF_FC
23. Afraid to go	1. Yes w/o prompt	2. Yes on prompt	3. No	RF_AG
24 Sought for alternative treatment	1. Yes w/o prompt	2. Yes on prompt	3. No	RF_AT
25. Other	1. Yes w/o prompt	2. Yes on prompt	3. No	RF_OT

★***This is the end of the interview. Thank you very much for your cooperation. If you have any questions, please do not hesitate to ask now or anytime.***

Time that this interview ends: ☐☐ : ☐☐
